# Phase evolution and property development in kaolin ceramics based on soda lime silica and granite sludge wastes

**DOI:** 10.1038/s41598-025-29504-2

**Published:** 2025-12-10

**Authors:** S. E. Abo Sawan, R. M. Khattab, Momen M. Ali, H. K. Abd El-Hamid

**Affiliations:** 1 Refractories, Ceramics and Building Materials Department, National Research Centre (NRC), El-Buhouth St., Dokki, Cairo, 12622 Egypt; 2 Construction Engineering and Management Department, Faculty of Engineering, Pharos University, Alexandria, Egypt

**Keywords:** Kaolin, Soda lime Silica, Granite sludge, Electrical, Dielectric properties, Chemistry, Engineering, Materials science, Nanoscience and technology

## Abstract

Soda lime silica (SLS) glass waste and granite sludge (GS) waste have been studied as eco-friendly raw materials in ceramic production. Different percentages of SLS or GS (10–30 weight percentage) are added to kaolin powder, pressed, and then sintered at 900, 1000, and 1100 °C. The goal of this research is to comprehend how waste content affects the electrical and physico-mechanical characteristics of ceramics made of silicate. X-ray fluorescence was used to characterize the starting materials (XRF). SEM and X-ray diffraction (XRD) are used to examine the effects of SLS or GS addition on microstructure and phase composition, respectively. Additionally examined are electrical and dielectric characteristics, bulk density, compressive strength, and apparent porosity. Results revealed that GS-containing samples are more porous than SLS-containing samples. Apparent porosity reaches 16.26% after 30 weight percentage of GS addition, while it is 6.50% for 30 weight percentage of SLS addition after sintering at 1100 °C. Maximum compressive strength (180 MPa) is obtained at 1100 °C for the sample containing 10 weight percentages of GS. However, the electrical and dielectric properties are enhanced by SLS addition due to the excellent alkali ionic mobility in the matrix.

## Introduction

Global manufacturing of ceramic bodies has increased recently, raising concerns about the gradual depletion of basic materials. These factors have led the research community to look for new resources that meet the requirements of the ceramics industry. For instance, they investigate the feasibility of recycling and integrating industrial waste into different ceramic formulations^1–3^. Recycling these waste products helps lessen pollution and the depletion of natural raw materials. Various industrial wastes have created numerous kinds of ceramics in this context. These comprise glass waste, fly ash, red mud, solid ceramic waste, blast furnace slag, and glass frit^4–7^.

Utilizing solid waste has a greater effect on society, the environment, and human health. Utilization of solid wastes has two benefits: it reduces disposal issues and preserves natural raw materials. The ceramic body serves as an ideal matrix for combining various types of waste materials because of their diverse characteristics, utilizing a sintering process at a comparatively high firing temperature^8–10^. Granite sludge (GS) and soda lime silica (SLS) waste are two solid wastes used to manage this job. They are used to compare the impact of soda lime silica content or grain sludge to kaolin ceramic bodies in a laboratory setting to reduce water absorption, improve mechanical properties, and minimize linear shrinkage^11–14^

SLS waste was used with clay as a fluxing agent to reduce the softening point during the sintering of ceramic bodies. Due to their comparable chemical composition, soda lime silica glass has recently been discovered as an additional material to replace feldspar as a flux, and it makes sense to look into this. Originating from recycled materials, SLS has a melting point of approximately 726 °C^15,16^, comparable to feldspar’s melting point of roughly 1140–1150°C^17,18^. Nevertheless, the quantity of SLS used to replace feldspar in stoneware bodies must be such that it develops a certain level of verification. The sample becomes extremely glassy and brittle if ceramic bodies contain too much soda lime silica. Additionally, replacing 6% of the feldspar in the porcelain stoneware tile body with SLS waste reduces plastic deformation, which helps to seal pores and lower water absorption to 0.2% at 1080°C^19–22^. Owoeye et al.^23^ found that porcelain ceramics made from residual soda-lime glasses had more encouraging overall results, suggesting that soda-lime glass promotes better mullite growth and increases the creation of glassy phases. Sagirul Islam et al.^24^ discovered that partially substituting soda lime silica glass for clay enhanced the light expanded clay aggregate’s basic characteristics, including bulk density, porosity, water absorption, and compressive strength.

Among the most often utilized rock types, granite serves various functions. Its great strength allows it to be used as a building material. The quarrying process generates granite sludge (GS) waste, particularly during the sawing and polishing steps. A lot of water is utilized in machinery for lubrication and cooling. The quantity, size, and kinds of sawed blocks produced annually vary from factory to factory regarding water use. Some factories added iron powder and slaked lime to enhance the sawing blade’s lifespan and improve the sawing process: sludge, or granite slurry, results from mixing water and fine particles. In general, the main constituents of GS are feldspar, quartz, and trace amounts of mica and limestone^25,26^.

Numerous studies have examined these wastes in various industrial settings, including the production of bricks, concrete, cement, and ceramics. To lessen the detrimental effects of waste on the environment, it must be removed economically^25–27^. Nagaraj et al. looked at the possibility of employing granite sludge in a variety of amounts, from 0 to 60%, as a partial replacement for sand in the production of compacted stabilized earth blocks (CSEBs)^28^. Various engineering attributes were assessed, including water absorption, durability, and compressive strength. They found that the CSEBs’ engineering properties are enhanced by the addition of granite sludge, with less than 10% water absorption and a wet compressive strength between 3.5 and 7.8 MPa^28^. The viability of utilizing leftover granite and marble sawing waste to create bricks was examined by Haftu^29^. The constructed bricks’ chemical composition, plasticity, particle size, and petrological and mineralogical analyses were all evaluated. By adding as much as 50% weight to the initial clay material, they found that these wastes can enhance the bricks produced. In recent years, recycling GS waste in the ceramic industry has become a technological focus due to the potential to reduce costs of production, use residues as a secondary raw material to create very stable glassy phases (glass and glass–ceramic industry), and solve some problems in brick and tile production by incorporating granite wastes into their formulations^30^. Consistent with earlier research, this study examines how different firing temperatures affect the varied proportions of granite sludge or soda lime silica on kaolin powders. Additionally, it contrasts how the structural, mechanical, electrical, and dielectric characteristics of the resulting ceramic bodies are affected by soda lime silica and granite sludge waste.

## Materials and methods

### Preparation of the starting materials

In the development of this research, conventional ceramic kaolin raw material, soda lime silica glass, and granite sludge wastes were used. Kaolin is provided by Sinai Manganese Company, Sinai Peninsula, Egypt. Granite sludge was selected and collected from process industries located at Shaq Al-Thu’ban cluster, south of Cairo, Egypt. Soda lime silica glass waste is supplied by Crystal Asfoor Company, Shobra El Khema, Cairo, Egypt.

Kaolin, soda lime silica (SLS), and granite sludge (GS) were ground extremely finely until they passed through a 40 µm sieve in an electric milling machine (Retsch GmbH PM100, Germany). For homogenization, kaolin was mixed with different weight percentages (10–30 wt%) of SLS or GS. The composition details are presented later in Table 2. The mixture was then dry-milled with zirconia balls at 250 rpm for 30 min. The sample was formed using a mold of 1.25 cm diameter and pressed under a pressure of 40 MPa^30,31^. In an electrical furnace (Nabertherm), samples were sintered at temperatures of 900, 1000, and 1100 °C at a rate of 5 degrees Celsius per minute, with a soaking period of two hours for each temperature.

### Characterizations

- Quantitative analysis of the representative starting materials was performed using wavelength dispersive X-ray fluorescence (WD-XRF) with an AXIOS Sequential Spectrometer (Panalytical, 2005).
- X-ray diffraction (XRD) analysis was performed using a BRUKER AXS D8 ADVANCE diffractometer (Germany) to identify the crystalline phases formed in the prepared samples. The measurements were conducted under standard temperature and pressure conditions, using Cu Kα radiation operated at 45 kV and 40 mA, with a scanning range of 5°–70° (2θ) and a step size of 0.02°, with a counting time of 2 s per step^31^.
- Fourier Transform Infrared Spectroscopy (FTIR) analysis was conducted to investigate the infrared spectra of the samples. After preparation, the materials were finely ground, mixed with KBr in a 1:100 ratio, and pressed under a load of 5 tons/cm^2^ to produce a uniform, transparent pellet^32^.
- Thermogravimetric Analysis (TGA) was carried out using a LINSEIS STA PT1600 instrument. Each powdered sample was heated at a rate of 10 °C/min up to 1000 °C, and the corresponding weight changes and thermal behaviors were recorded in the resulting thermograms^30^.
- The particle size of the powders was assessed using energy-dispersive X-ray spectroscopy (EDS) in combination with a JEOL JEM-2100 transmission electron microscope (TEM), which was equipped with a high-angle annular dark field detector.
- A scanning electron microscope (SEM), specifically the FEI QUANTA FEG 250, was employed to investigate the microstructure of the fired samples.
- Prior to immersion in water, the saturated samples were first weighed in air (Ws) and then reweighed while submerged (Wi). After drying the samples at 110 °C overnight, their dry weight (Wd) was recorded. Using the specific gravity of water (γ), the apparent porosity (AP) and bulk density (BD) of the samples were calculated using the following equations:^31–33^.
  1 $${\text{AP}} = \left[ {{\text{WS}} - {\text{WD}}/{\text{WS}} - {\text{WI}}} \right] \, *{1}00$$
  2 $${\text{BD}} = \left[ {{\text{WD}}/{\text{WS}} - {\text{WI}}} \right] \, *\gamma$$
- The compressive strength of the fired specimens was determined using a Tinius Olsen Universal Testing Machine (Model 25ST, UK) operating at a crosshead speed of 0.5 mm/min. The evaluation of cold crushing strength (CCS) followed the ASTM C 1424–19 standard for samples with an area of 1.23 cm^2^ and a length of 1.5 cm.
- An impedance analyzer (YHP4192A, Yokokawa, Hewlett-Packard Japan Ltd., Tokyo, Japan) was used to assess the electrical properties, including conductivity, dielectric loss, and dielectric constant. Measurements were conducted over a frequency range of 0.1 Hz to 20 MHz. To ensure accuracy, five measurements were taken for each parameter: bulk density, apparent porosity, compressive strength, and electrical properties.

## Results and discussion

### Evaluation of the beginning materials

Table 1 presents the initial materials’ XRF results. Silica and alumina make up the majority of granite sludge, with trace levels of other oxides such as Na_2_O, Fe_2_O_3_, CaO, and K_2_O. According to the chemical analysis, soda lime silica has a greater alkali content (Na_2_O, CaO, MgO, and K_2_O). About 26.14% of soda lime silica is composed of Na_2_O + CaO + MgO. The fluxing agents’ content (11.98 weight percent = Na_2_O + CaO + MgO + K_2_O) should encourage the liquid phase formation^34^. Table 1 further shows that the percentages of kaolin that contain SiO_2_ and Al_2_O_3_ are 50.01% and 32.87%, respectively. Furthermore, it still includes a lot of impurities, including K_2_O, Fe_2_O_3_, TiO_2_, MgO, and SO_3_. Because of organic materials and volatile components, there is an approximate 12.59% loss upon fire^35,36^. The composition of the mixed samples is presented in Table 2.

**Table 1:** XRF Analysis of starting materials in wt.%.

Oxide in wt.,%	Starting materials		
	Kaolin	granite sludge (GS)	Soda lime silica (SLS)
SiO_2_	50.01	62.77	71.20
Al_2_O_3_	32.87	14.92	0.66
MgO	–	1.79	4.03
Fe_2_O_3_	1.54	6.76	0.10
CaO	0.24	3.19	7.05
Na_2_O	0.22	4.69	15.01
K_2_O	–	2.31	0.05
P_2_O_5_	0.15	0.70	0.03
SO_3_	0.39	0.19	0.15
TiO_2_	1.57	0.83	0.02
Cl	0.09	0.15	0.04
MnO	–	0.02	–
NiO	0.029	0.009	–
CuO	–	0.021	–
ZnO	0.048	0.016	–
SrO	0.033	0.041	0.006
Rb_2_O	–	0.007	–
ZrO_2_	0.125	0.058	0.017
Ga_2_O_3_	0.012	0.005	–
Y_2_O_3_	0.012	–	–
V_2_O_5_ Nb_2_O_5_ Cr_2_O_3_	0.058 0.020 –	– 0.006 –	– – 0.005
LOI	12.59	1.25	1.66

**Table 2:** The Mix composition of the prepared samples in wt.%.

Sample code	Mixed samples in wt%			Mixture’s chemical constituents in wt%									
	Kaolin	Soda lime silica (SLS)	Granite Sludge (GS)	SiO_2_	Al_2_O_3_	MgO	Fe_2_O_3_	CaO	Na_2_O	K_2_O	P_2_O_5_	Cl	SO_3_
Kaolin	100	0	0	50.01	32.87	0	1.54	0.24	0.22	0	0.15	0.09	0.39
10% SLS	90	10	0	52.12	29.64	0.403	1.396	0.921	1.699	0.005	0.138	0.085	0.366
20% SLS	80	20	0	54.25	26.43	0.806	1.252	1.602	3.17	0.010	0.126	0.080	0.342
30% SLS	70	30	0	56.36	23.21	1.209	1.11	2.283	4.657	0.015	0.114	0.075	0.318
10% GS	90	0	10	51.28	31.07	0.179	2.062	0.535	0.667	0.231	0.205	0.096	0.370
20% GS	80	0	20	52.56	29.28	0.358	2.584	0.830	1.114	0.462	0.260	0.102	0.350
30% GS	70	0	30	53.83	27.48	0.537	3.106	1.125	1.561	0.693	0.315	0.108	0.330

**Fig. 1:**
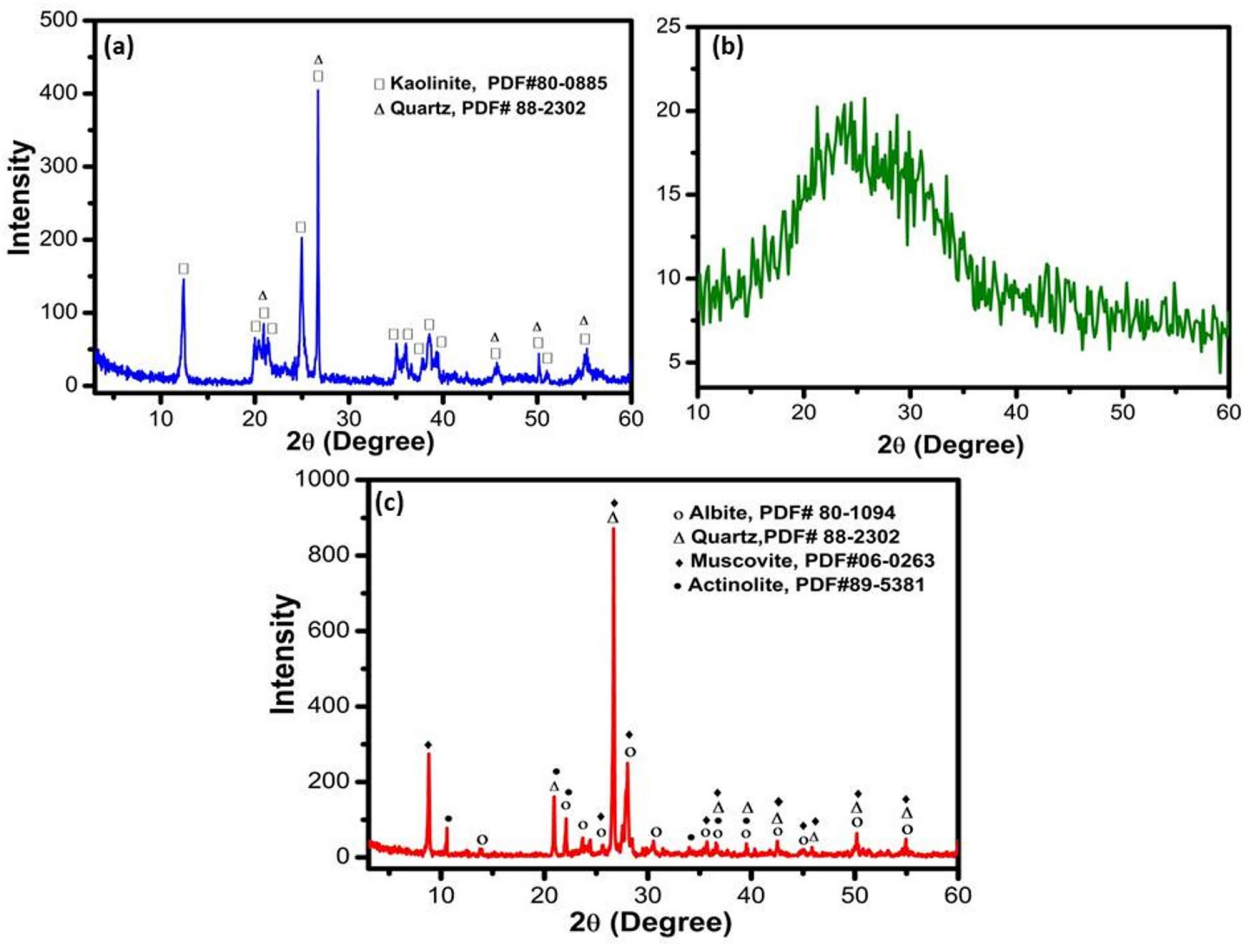
XRD patterns of (**a**) kaolin, (**b**) soda lime silica (SLS), and (**c**) granite sludge (GS).

The XRD patterns of kaolin, SLS, and GS wastes are presented in Fig. 1a, b, and c, respectively. As identified by the reference cards PDF# 80–0885 and PDF# 88-2302, the kaolin sample (Fig. 1a) exhibits characteristic diffraction peaks corresponding to kaolin and quartz phases. In contrast, the SLS sample (Fig. 1b) shows the absence of distinct diffraction peaks, confirming its amorphous nature. Meanwhile, the XRD pattern of granite sludge (GS) (Fig. 1c) reveals the presence of several crystalline phases, including actinolite (PDF# 89-5381), muscovite (PDF# 06-0263), quartz (PDF# 89-1961), and albite (PDF# 89-6430), indicating its heterogeneous mineral composition.

**Fig. 2:**
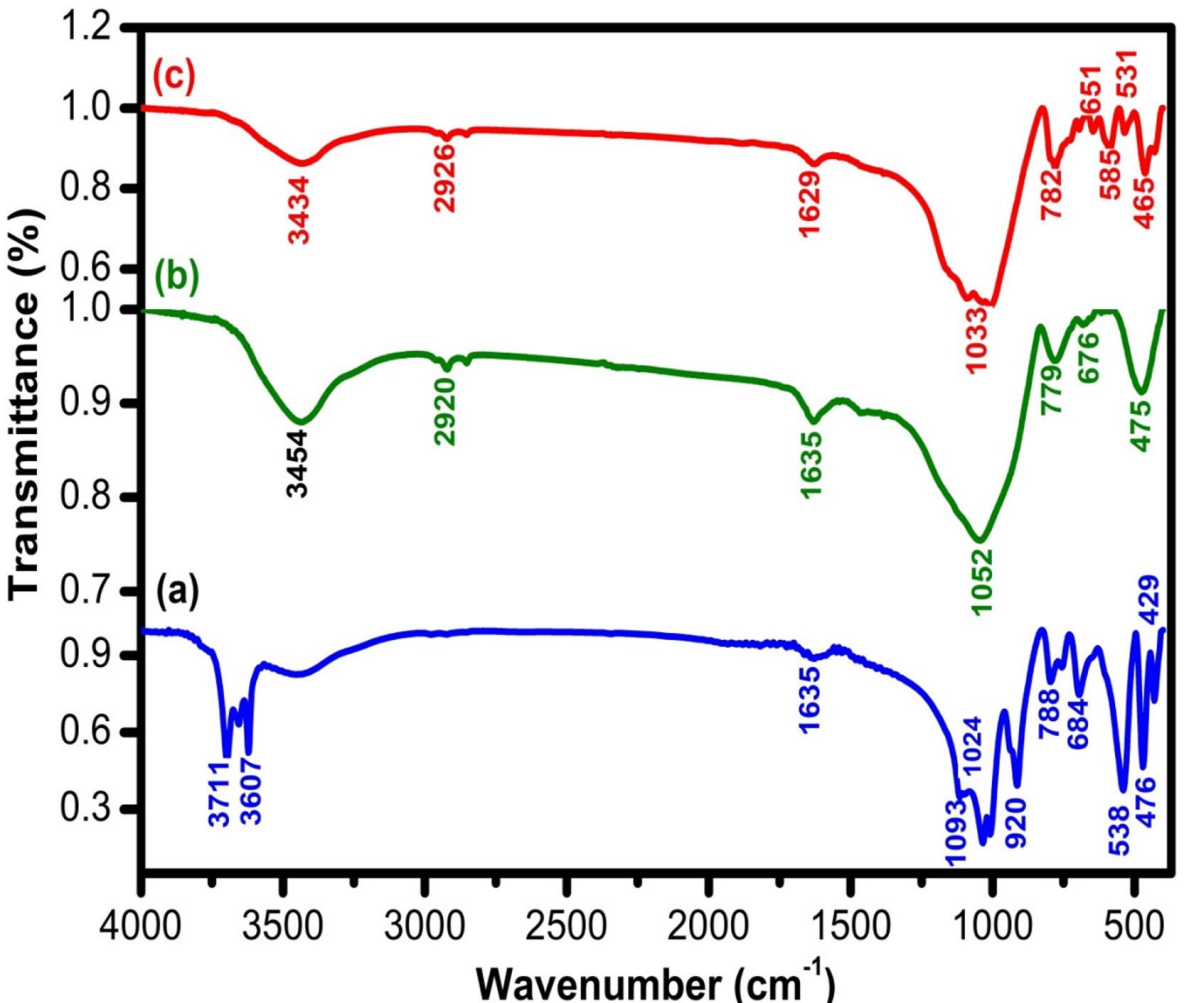
FTIR spectra of (**a**) kaolin, (**b**) soda lime silica (SLS), and (**c**) granite sludge (GS).

FTIR spectra of kaolin, GS, and SLS wastes are displayed in Fig. 2a, b, and c, respectively. The FTIR spectrum of kaolin (Fig. 2a) exhibited characteristic absorption peaks at 3711 cm^−1^ and 3607 cm^−1^, corresponding to O–H stretching vibrations, as well as the band at 1635 cm^−1^ is related to the molecular water. The bands observed at 1093 cm^−1^ and 1024 cm^−1^ were assigned to Si–O stretching in SiO₄ groups. Additionally, Si–O–Si bending vibrations were identified in the 429–481 cm^−1^ range.^37^, while the band at 920 cm^−1^ was attributed to Al(IV)–OH vibrations. Peaks at 788 cm^−1^ and 684 cm^−1^ were associated with symmetric Si–O stretching. The absorption at 538 cm^−1^ was linked to Si–O–Al(VI) vibrations, with Al in octahedral coordination^38^.

The main absorption bands observed in the FTIR spectrum of SLS (Fig. 2b) correspond to characteristic vibrational modes of silicate structures. The bands at 475 and 676 cm^−1^ are typically associated with Si–O–Si and O–Si–O bending vibrations, respectively. Additionally, the band appearing at 779 cm^−1^ is attributed to the symmetric stretching of Si–O–Si bonds involving bridging oxygen atoms between tetrahedra, whereas the band at 1052 cm^−1^ is linked to the antisymmetric stretching of Si–O–Si within the tetrahedral framework^39^. A band at 1635 cm^−1^ can be ascribed to molecular water or hydroxyl-related vibrations, indicating the presence of adsorbed moisture. Furthermore, the absorption band at 2920 cm^−1^ corresponds to symmetric stretching modes of interstitial H_2_O molecules, while the broad feature near 3554 cm^−1^ arises from molecular water, confirming residual hydration within the glass matrix^40^.

The FTIR spectrum of the granite sample (Fig. 2c) exhibits several characteristic absorption bands corresponding to silicate structures. Peaks appearing in the ranges of 465–531 cm^−1^, 585–651 cm^−1^, and at 782 cm^−1^ are attributed to ring vibrations of silicates, confirming the presence of quartz^41^. In addition, the prominent band at 1033 cm^−1^ corresponds to typical silicate vibrations, mainly associated with the stretching modes of Si–O or Al–O bonds within the mineral framework. The bands observed at 1629 cm^−1^ and 2926 cm^−1^ are related to molecular water or atmospheric moisture, suggesting slight hydration on the surface. Moreover, the absorption peak at 3434 cm^−1^ arises from O–H stretching vibrations, which can be linked to Al–OH and Si–OH groups, indicating the presence of hydroxyl functionalities in the granite sample^42^.

**Fig. 3:**
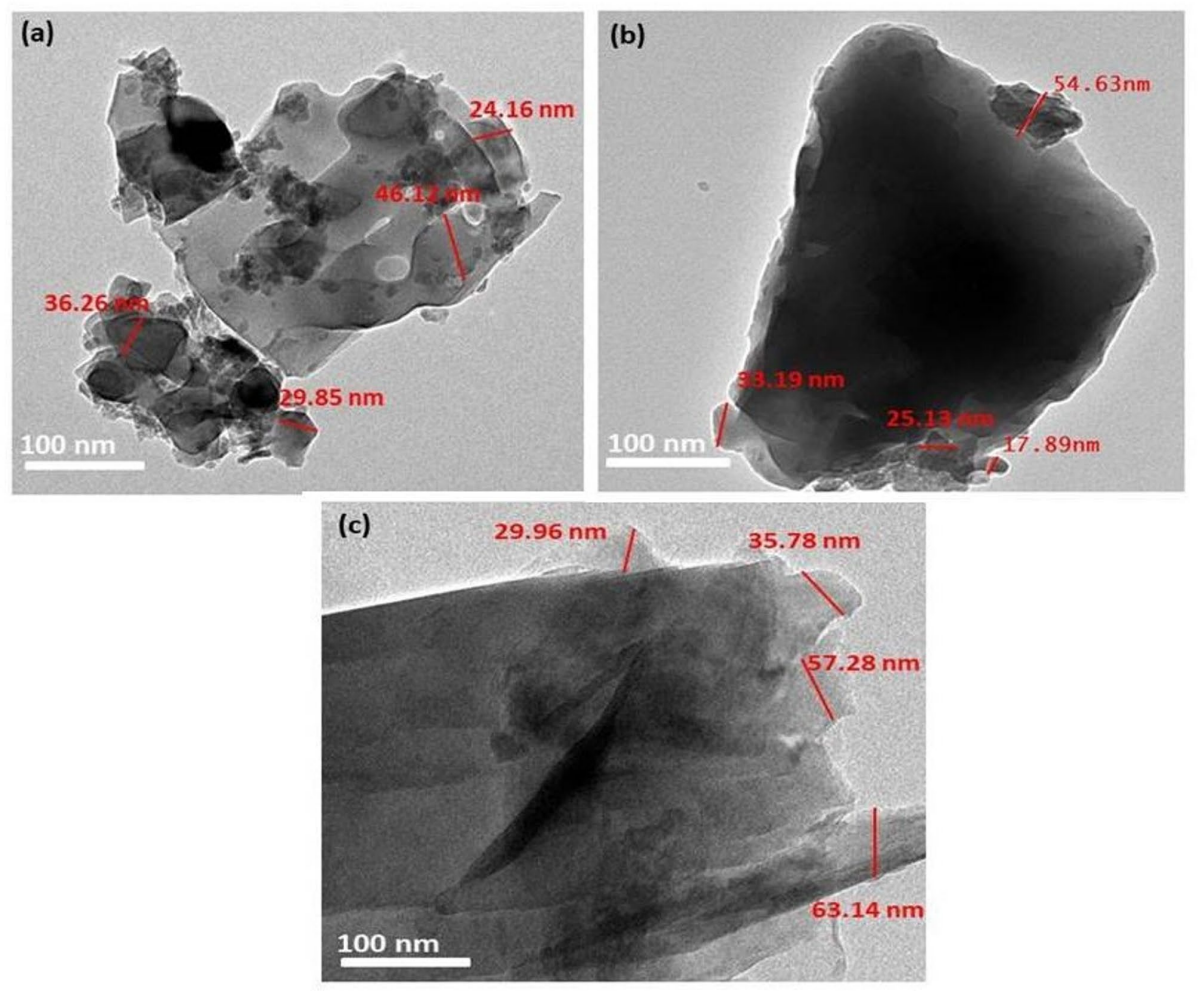
TEM images of (**a**) kaolin, (**b**) soda lime silica (SLS), and (**c**) granite sludge (GS).

TEM images of kaolin, SLS, and GS are shown in Fig. 3 a, b, and c respectively. As can be shown in Fig. 3a, the particle size of kaolin ranges from 24.16 to 46.12 nm, and the particle size of SLS ranges from 17.89 to 54.63 nm (Fig. 3b), whereas the particle size of GS (Fig. 3c) ranges from 29.96 to 63.14 nm.

**Fig. 4:**
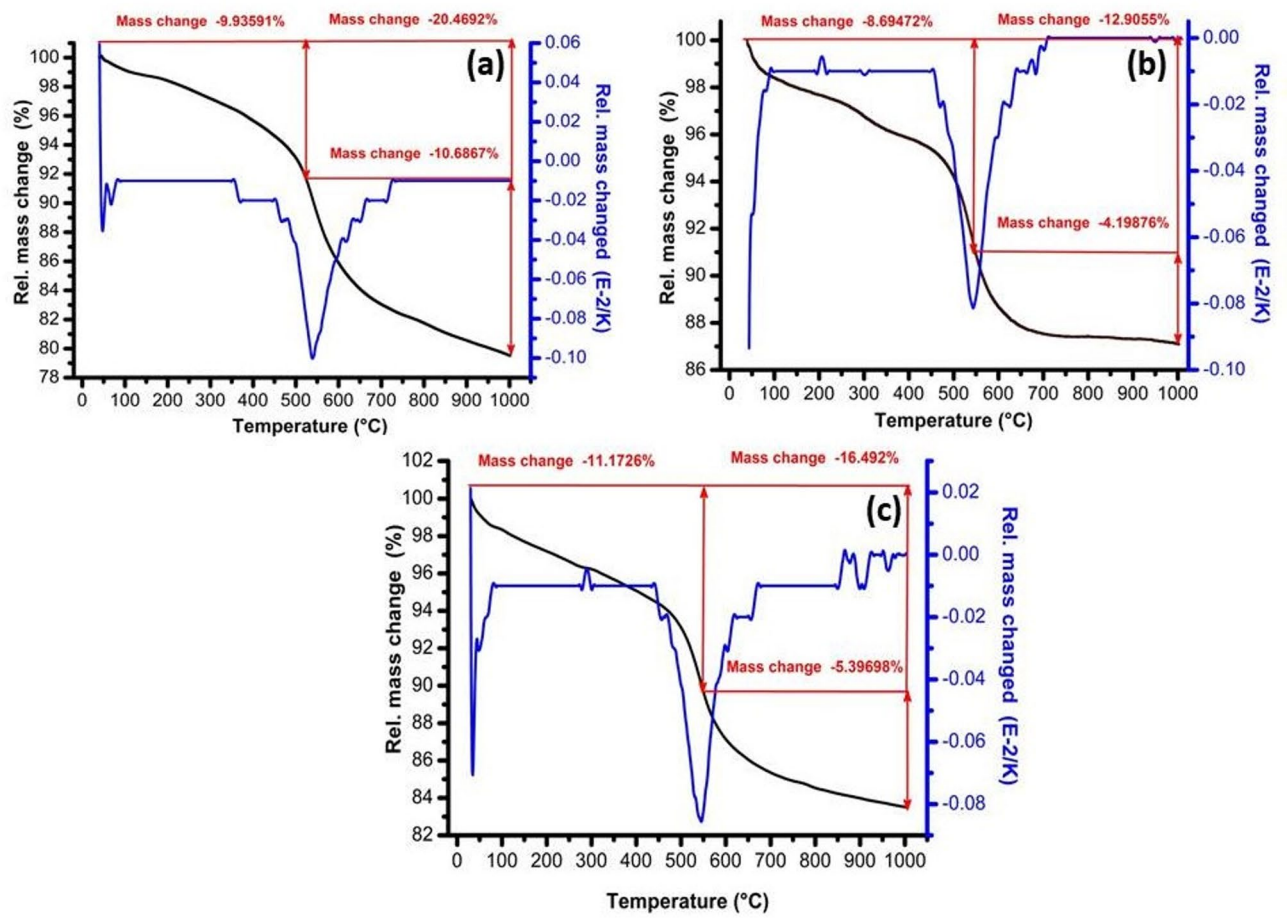
TGA of the green sample composed of (**a**) kaolin, (**b**) 30% SLS, and (**c**) 30% GS samples.

The thermal behavior of the green sample composed of kaolin, 30% SLS, and 30% GS was analyzed using TGA equipment, as illustrated in Fig. 4a, b, and c, respectively. The TGA curve of kaolin (Fig. 4a) shows an initial weight loss of approximately 0.6% below 200 °C, which can be attributed to the removal of physically adsorbed moisture. A subsequent weight loss of about 9.3% occurs between 300 °C and 400 °C, accompanied by a peak in the rate of mass change at 361.9 °C, corresponding to the release of bound water from the crystal structure. As the temperature continues to rise, a pronounced weight loss of 10.6% is observed around 537.6 °C, associated with dehydroxylation of the kaolinite interlayers^43^. In comparison, the total weight loss for the kaolin sample containing 30 wt.% SLS (Fig. 4b) is reduced to 12.9%. This decrease in mass loss is attributed to the formation of a liquid phase from the soda-lime component, which facilitates the dissolution of solid particles and the subsequent growth of crystalline phases such as mullite^44,45^. The TGA profile of kaolin containing 30 wt.% GS (Fig. 4c) exhibits a similar thermal behavior to that of pure kaolin. However, the total weight loss increases to 16.4%, which can be attributed to the oxidation of organic residues and the evaporation of structural (crystalline) water. Additionally, a further mass reduction occurs near 580 °C, corresponding to the decomposition of calcite^46^, followed by the thermal decomposition of granite minerals such as orthoclase^47^.

### Characterization of sintered samples

#### Physical properties of the sintered samples

**Fig. 5:**
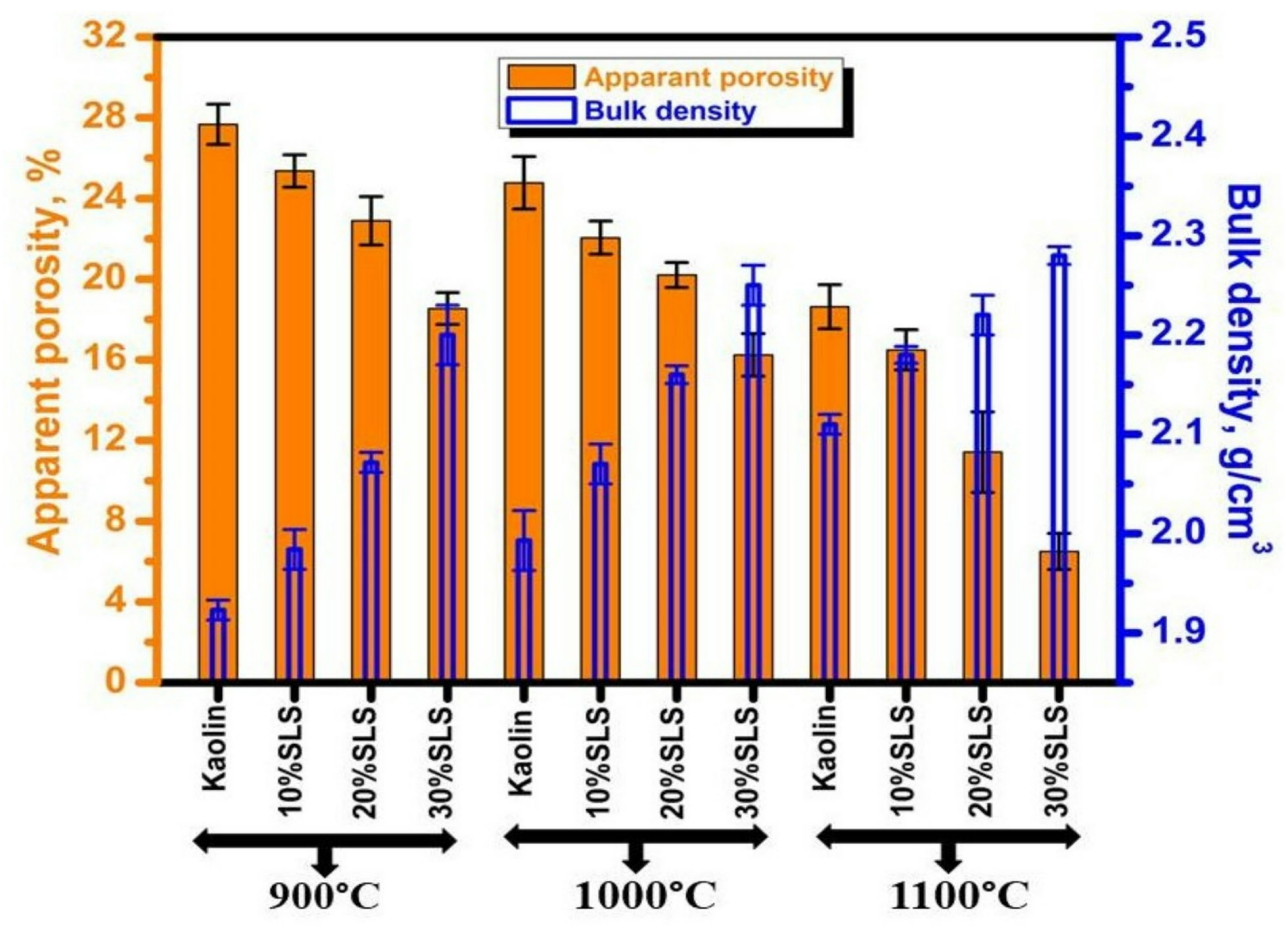
Physical properties of burned ceramics at 900, 1000, and 1100 °C with different proportions of SLS.

**Fig. 6:**
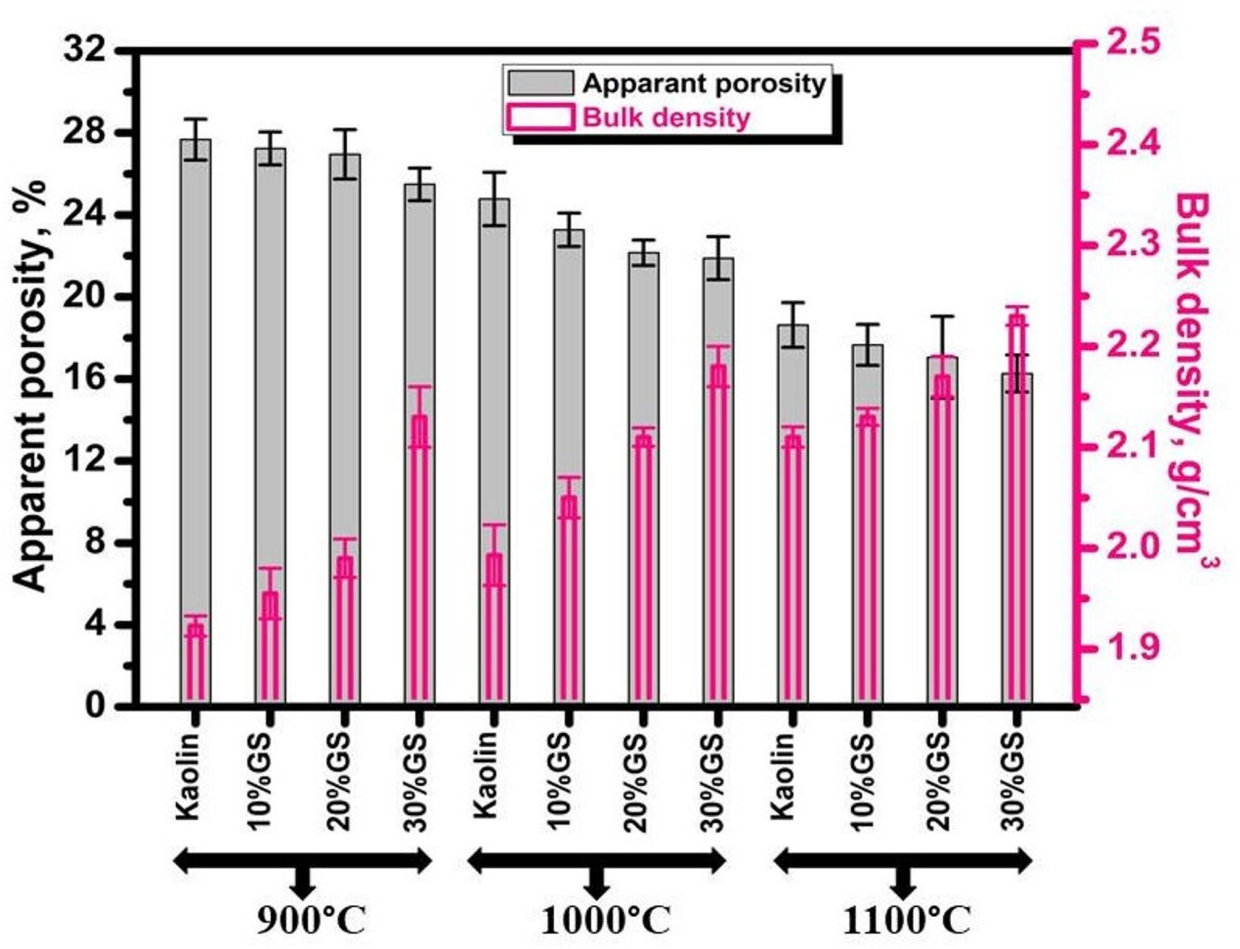
Bulk density and apparent porosity of burned ceramics at 900, 1000, and 1100 °C with different proportions of GS.

Figures 5 and 6, along with Table 3, illustrate the variations in bulk density and apparent porosity of ceramics fired at 900, 1000, and 1100 °C, each containing different amounts of SLS and GS, respectively. In general, increasing the firing temperature led to a gradual rise in bulk density and a corresponding reduction in apparent porosity for both kaolin–SLS and kaolin–GS compositions. Nevertheless, the increase in bulk density was less pronounced than that observed for pure kaolin fired at 1100 °C. The apparent porosity of the fired kaolin ceramics, initially ranging between 27.67% (at 900 °C)% and 18.63% (at 1100 °C), decreased significantly upon the addition of SLS to the ceramic mixture. For example, samples containing 30 wt% SLS exhibited a notable reduction in porosity, from 18.54% (at 900 °C) to 6.50% (at 1100 °C) (Fig. 5), accompanied by an increase in bulk density from 2.20 (at 900 °C) to 2.28 g/cm^3^ (at 1100 °C). The improvement in densification can be attributed to the fluxing oxides present in SLS, primarily CaO and Na_2_O, which promote the formation of a liquid phase during firing. This liquid phase infiltrates the interparticle spaces, filling the voids and transforming open pores into closed ones. Consequently, the overall volume of the sample decreases, leading to increased linear shrinkage and reduced water absorption^44,48^. These results confirm the strong interdependence between densification behavior and microstructural characteristics such as linear shrinkage, porosity, and water absorption^44,49^.

**Table 3:** Apparent porosity and bulk density of kaolin ceramics with different proportions of SLS or GS at 900, 1000, and 1100 °C.

Firing Temp., °C	Sample Code	Apparent porosity, %	+ SD	Bulk density, g/cm^3^	+ SD
900	Kaolin	27.67	1	1.923	0.01
	10% SLS	25.36	0.8	1.984	0.02
	20% SLS	22.89	1.2	2.071	0.01
	30% SLS	18.54	0.79	2.20	0.03
	10% GS	27.24	0.79	1.955	0.025
	20% GS	26.95	1.19	1.99	0.019
	30% GS	25.49	0.76	2.13	0.029
1000	Kaolin	24.77	1.3	1.993	0.03
	10% SLS	22.05	0.82	2.07	0.02
	20% SLS	20.2	0.61	2.16	0.009
	30% SLS	16.24	1.05	2.25	0.02
	10% GS	23.27	0.78	2.05	0.018
	20% GS	22.15	0.61	2.11	0.008
	30% GS	21.89	1.045	2.18	0.024
1100	Kaolin	18.63	1.09	2.11	0.01
	10% SLS	16.49	1.01	2.18	0.0085
	20% SLS	11.42	2	2.22	0.02
	30% SLS	6.50	0.9	2.28	0.009
	10% GS	17.66	1	2.13	0.0079
	20% GS	17.05	1.18	2.17	0.018
	30% GS	16.26	0.85	2.23	0.0089

Figure 6 shows the bulk density increase and open porosity decrease for samples with 10–30 weight percent GS sintered at 900, 1000, and 1200 °C. However, these samples’ densification characteristics are lower than those of SLS samples. This is due to the low flux in GS^50,51^. After adding 30 weight percent GS, the bulk density values vary from 2.13 (at 900 °C) to 2.23 g/cm^3^ (at 1100 °C), whereas the porosity is between 25.49 (at 900 °C) and 16.26% (at 1100 °C). Additionally, the high concentration of Fe_2_O_3_ in granite sludge, which breaks down at high temperatures and causes porosity to rise, may be the reason for these samples’ higher porosity than SLS^50,51^. Often, the waste’s higher concentration of fluxing agents (CaO, Na_2_O, and K_2_O) promotes the liquid phase’s development, hastening the sintering and densification of the ceramic bodies^52^. This results from a verification process that reduces porosity. Verification is a process that occurs when solid particles are involved in the creation of a liquid phase^35^. Particles are rearranged by surface tension and capillarity, which encourages the densification and contraction of the open pores^35,52,53^. The following characterizations are therefore performed at 1100 °C by these findings.

#### Phase structure

**Fig. 7:**
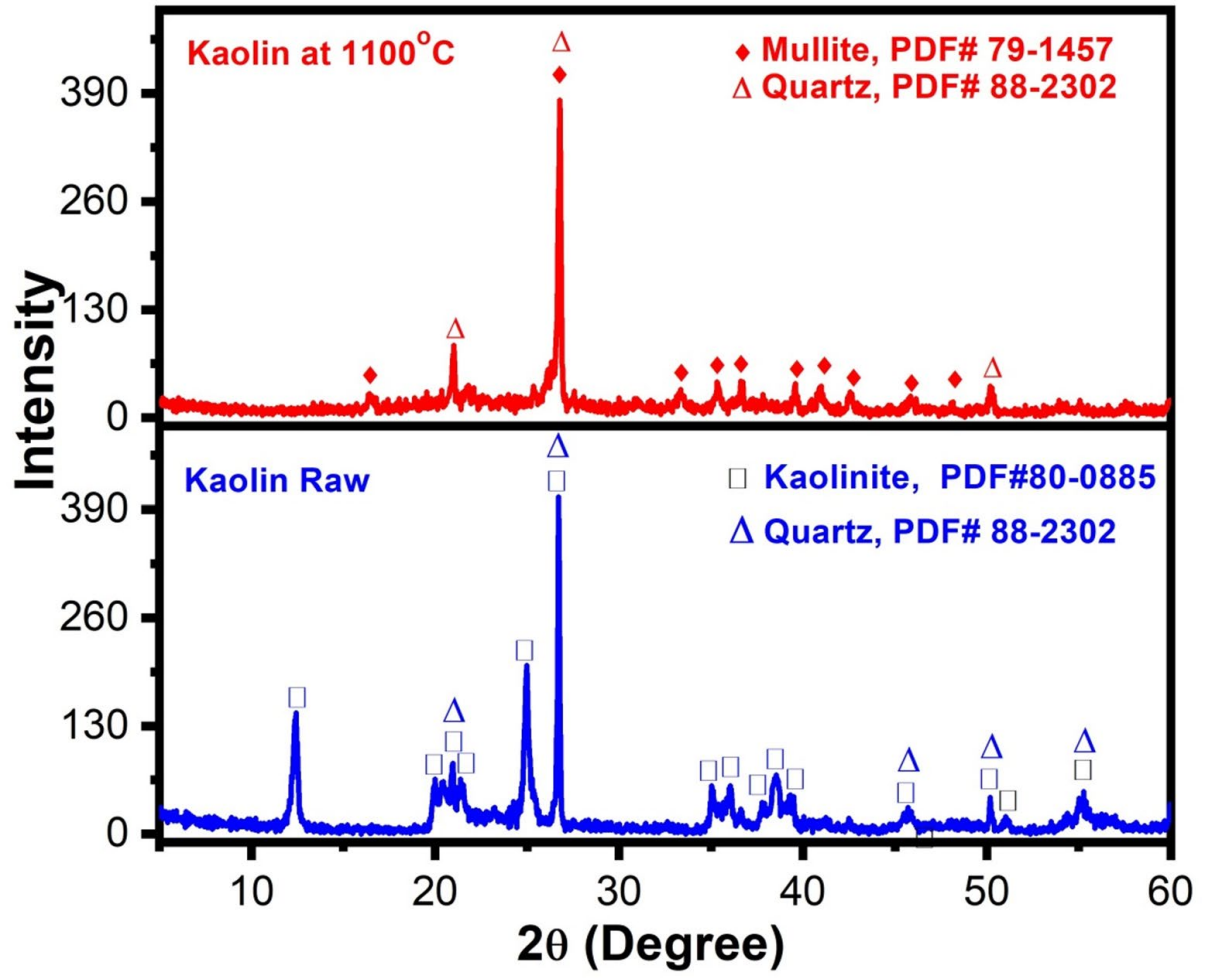
XRD pattern of the raw kaolin and the kaolin fired at 1100 °C.

Figure 7 compares the XRD patterns of raw (green) kaolin and kaolin heated to 1100 °C. The results indicate that thermal treatment significantly alters the crystal structure of kaolin. Variations in peak positions and intensities reveal the progressive transformation of kaolinite, the primary mineral in kaolin, into more thermally stable phases, mostly mullite, according to PDF#79-1457, upon firing at 1100 °C. This transformation enhances the crystallinity and heat resistance of the material^43^.

**Fig. 8:**
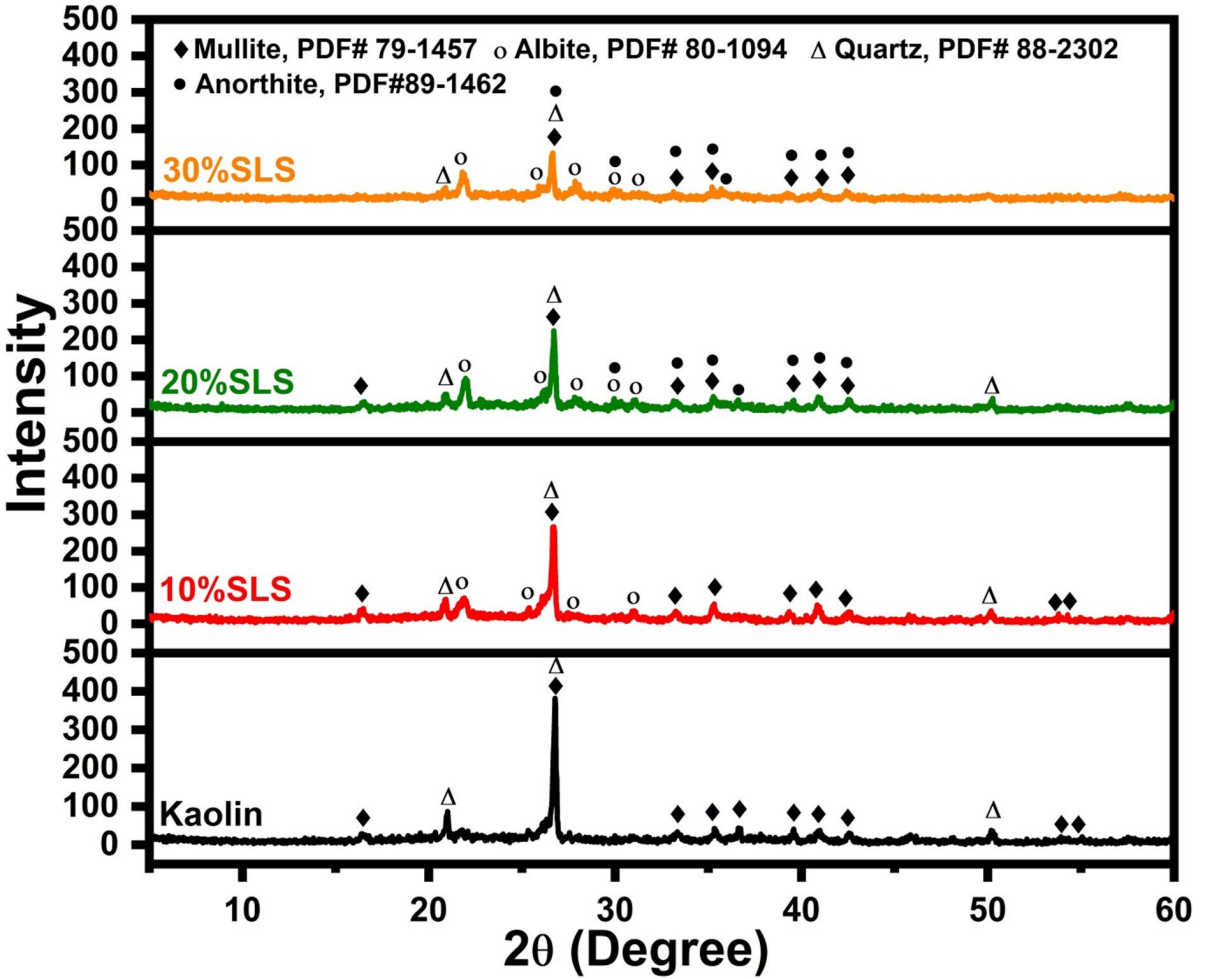
XRD patterns after adding 10 to 30 wt.% of SLS to kaolin fired at 1100 °C.

**Fig. 9:**
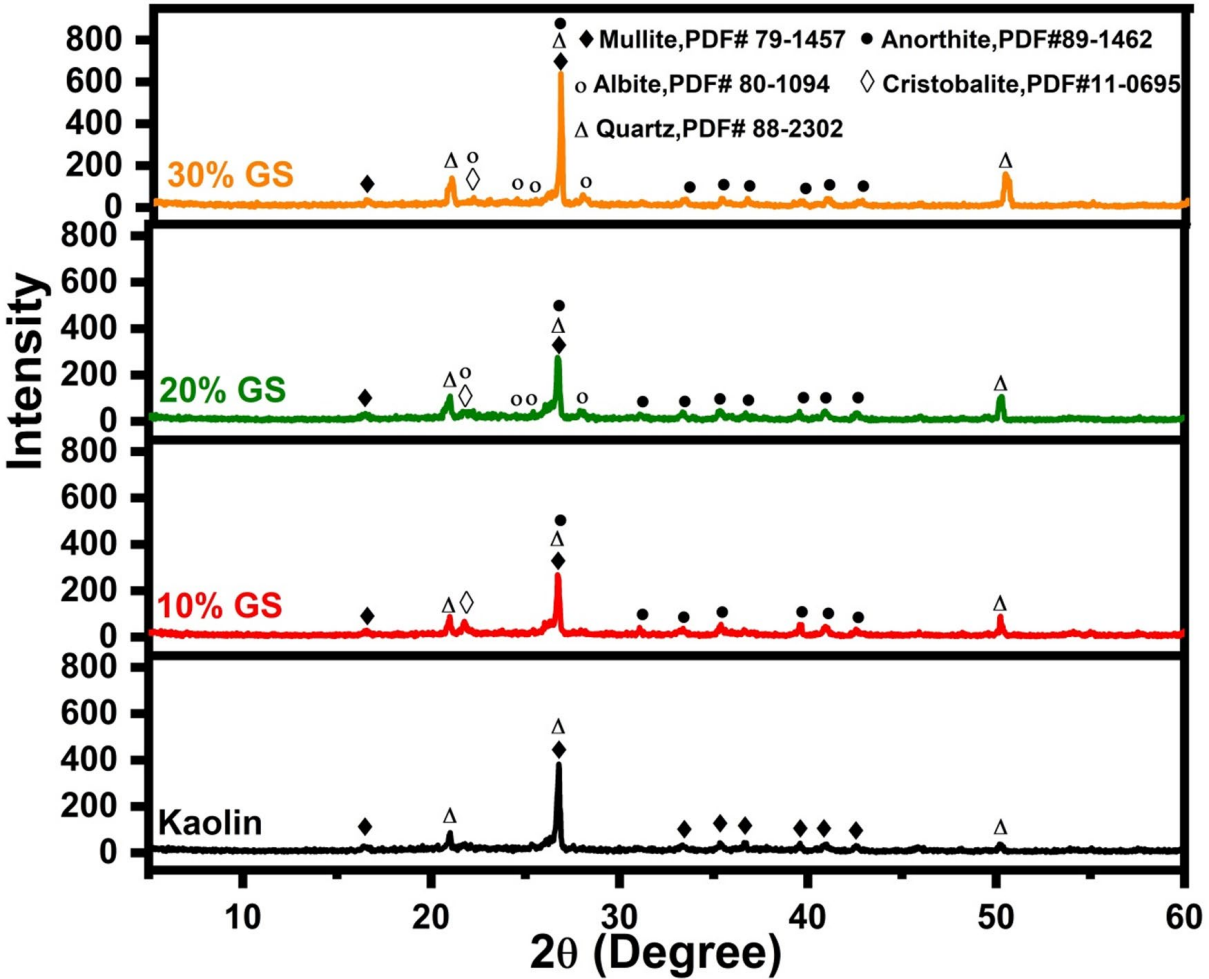
XRD patterns after adding 10 to 30 wt.% of GS to kaolin fired at 1100 °C.

Figures 8 and 9 display the XRD patterns of fired samples with varying percentages of SLS and GS at 1100 °C, respectively. Figure 8 shows that the samples include mullite (Al_6_Si_2_O_13_, PDF# 79-1457), quartz (SiO_2_, PDF# 88-2302), and albite (NaAlSi_3_O_8_, PDF#80-1094) with a small amount of anorthite (CaAl_2_Si_2_O_8_, PDF#89-1462). It is observed that the quartz content gradually decreases with increasing SLS addition. At the same time, the XRD patterns show that the quartz peaks shift toward higher 2θ values as the soda-lime content increases, which is attributed to the greater formation of the glassy phase. This glassy phase progressively dissolves the surface of the quartz particles, and the resulting reactions induce modifications in the lattice parameters and crystal structure of the remaining quartz, leading to slight shifts in its characteristic diffraction peaks. When kaolin is fired with a soda-lime flux, the observed shift in quartz peaks mainly arises from the formation of solid solutions and lattice strain. The soda-lime acts as a fluxing agent, reacting with alumina and silica in kaolin at elevated temperatures. This interaction causes part of the quartz (SiO_2_) to dissolve into the amorphous glassy matrix, altering the composition and structural order of the residual crystalline quartz, and consequently producing peak shifts in the XRD pattern. Moreover, aluminum (Al^3+^) and sodium (Na^+^) ions originating from the kaolin and soda-lime may enter the lattice of high-temperature quartz. The substitution of Al^3+^ for Si^4+^ introduces a charge imbalance that is compensated by the incorporation of Na^+^ ions. These substitutions modify the lattice parameters (a and c) of quartz, which, according to Bragg’s law, results in a shift of the diffraction peaks toward higher 2θ values. Thus, the combined effects of quartz dissolution, solid-solution formation, and ionic substitution explain both the reduction in quartz intensity and the observed peak shifts with increasing SLS content^54,55^.

Additionally, the amount of albite increases when the amount of SLS increases. The presence of albite in all samples originates from the crystallization of plagioclase, rather than being a direct product of the devitrification of the SLS glass particles. Since the initial SLS glass contains only 0.66 wt% Al_2_O_3_ (Table 1), the decomposed kaolinite serves as the sole source of alumina required for plagioclase formation. Therefore, the interface between partially melted SLS glass particles and meta-kaolinite is considered the most probable site where plagioclase crystallization begins^56^. The appearance of anorthite can be attributed to the calcium content in the glass waste, which promotes the reaction between CaO and metakaolinite (CaO + metakaolinite → anorthite)^57^. Each sample has a certain amount of mullite, the formation of mullite can be explained as follows: metakaolinite maintains its structure up to approximately 920 °C, and its decomposition begins near 940 °C. A spinel-type phase is formed not by the decomposition of metakaolinite but by a topotactic formation with respect to metakaolinite before its decomposition. As the breakdown of metakaolinite progresses around 940 °C, mullite formation is initiated while the spinel-type phase persists. Complete decomposition of this spinel-type phase occurs around 1200 °C, leading to the crystallization of cristobalite from amorphous silica and the pronounced growth of mullite crystals^58^.

However, samples containing waste glass exhibit a significantly lower proportion of mullite, particularly those with 30 wt% SLS. This observation can be explained by the effect of the high flux content in soda-lime silicate glass, which promotes the formation of a substantial glassy phase during firing. This glassy phase reacts with the alumina and silica components of kaolin, consuming part of the essential constituents required for mullite crystallization. As a result, mullite either fails to form or is significantly reduced. Moreover, soda-lime acts as a strong flux, lowering the melting temperature and generating a liquid phase that alters the chemical equilibrium of the system. This molten phase facilitates the dissolution of alumina and silica into the glassy matrix instead of forming crystalline mullite. Consequently, at higher SLS concentrations (such as 30%), the structure becomes predominantly glassy, leading to the disappearance of mullite peaks in the XRD pattern^56–58^. Generally, the amount of vitreous phase increases progressively with the addition of more soda-lime glass. The incorporation of soda-lime glass appears to shift the balance between the glassy and crystalline phases in kaolin, resulting in reduced crystallization. The liquid phase becomes saturated with SiO_2_, Al_2_O_3_, Na_2_O, and CaO, largely due to the significant presence of Na_2_O and CaO. This promotes more efficient melting of quartz while simultaneously reducing crystallization and/or causing partial dissolution of mullite. Consequently, this contributes to the stability of feldspar (albite) even at elevated temperatures^59^.

Figure 9 shows the XRD of the GS samples after they are fired at 1100 °C. In addition to the above phases in SLS, cristobalite (SiO_2_, PDF#11-0695) also appeared. The plagioclase (anorthite, albite) and quartz phases increased with GS. On the other hand, the mullite phase decreases when the amount of GS increases (Fig. 9). The progressive decrease in mullite phase intensity observed in all fired samples, along with the slight drop in quartz phase intensity associated with the appearance of the cristobalite phase up to a 10% GS addition, is likely due to the conversion of quartz into cristobalite^50,60^. However, further increasing the GS content by 10% results in a reduction in the cristobalite phase intensity. This could be because alkali oxides (Na_2_O and K_2_O) are added, delaying or even stopping cristobalite crystallization^50,61^.

Additionally, as the fluxing oxide content increased, as in SLS, a sufficient liquid phase developed to establish a vitrification process at a comparatively lower temperature, causing the quartz phase to gradually dissolve into the glassy phase^50,62,63^. In Fig. 9, this glassy phase is seen as a hump peak that rises with increasing GS and ranges from 20 to 30 (2θ). The presence of anorthite (or diopside) in the fired compositions and the reduction in mullite crystallization caused by GS addition imply that the SiO_2_-Al_2_O_3_-CaO system is dictating phase equilibrium^64^. This indicates that the collective effect of all alkaline and alkaline-earth fluxing oxides (K_2_O, Na_2_O, CaO, and MgO) should be considered in the phase equilibrium analysis, which is consistent with the results reported by Ozturk et al.^65^. So the addition of GS (low flux content) retains the stability of the quartz and inhibits its dissolution into the glassy phase. This is happening as a result of increased GS, which increases the amount of quartz.

#### FTIR analysis

**Fig. 10:**
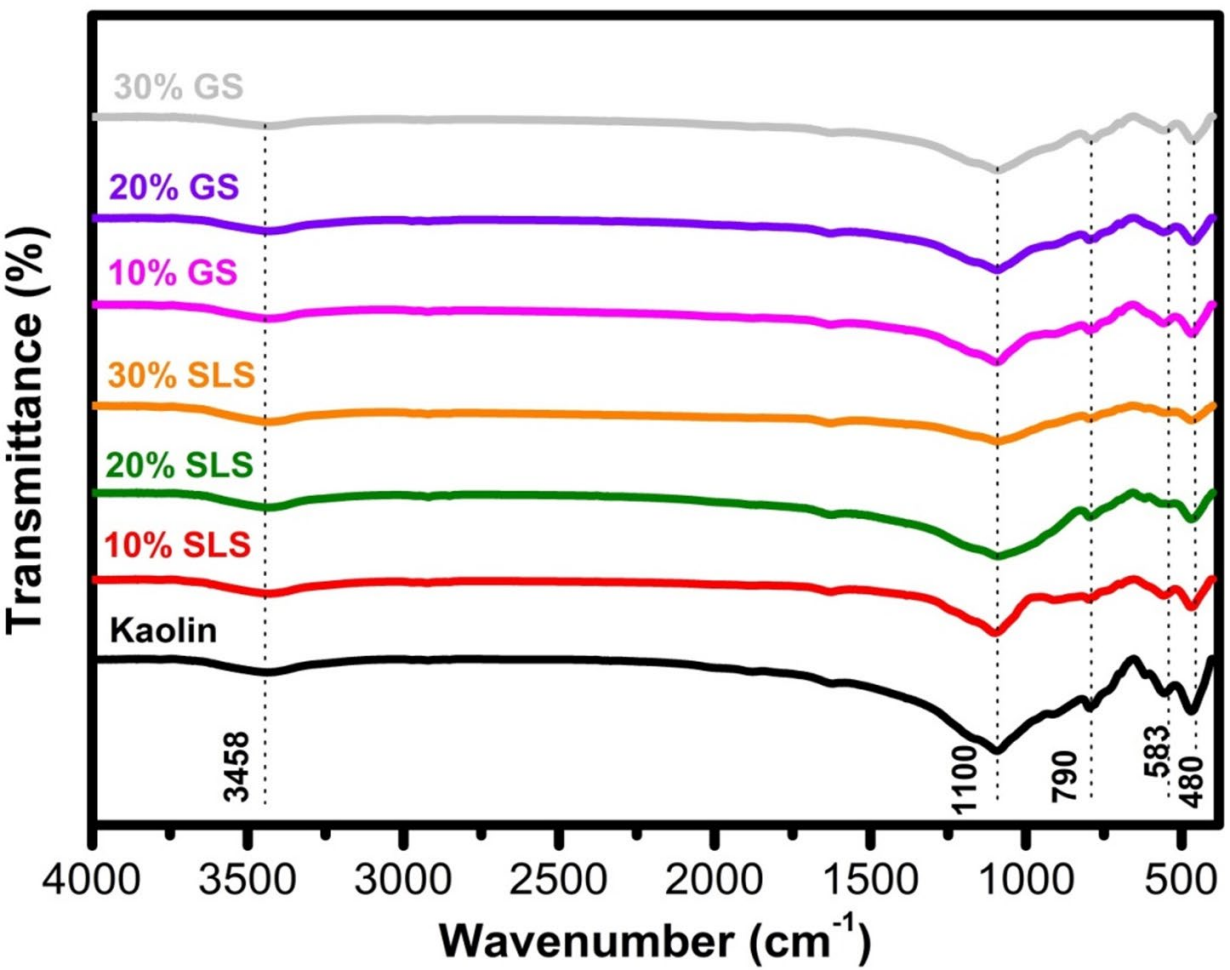
FTIR spectra after adding 10 to 30 wt.% of SLS or GS to kaolin fired at 1100 °C.

After thermally treating kaolin both with and without the addition of SLS and GS at 1100 °C for 2 h, as shown in Fig. 10, the hydroxyl (OH) vibration peaks at 3458 cm^−1^ and 1635 cm^−1^ were notably diminished, indicating a successful transformation into calcined kaolin. The band at 1100 cm^−1^, which corresponds to amorphous SiO_2_, was detected^38^. The absorption band associated with Al(IV)–OH vanished due to distortions within the tetrahedral and octahedral sheets of kaolinite. A new peak appeared at 790 cm^−1^, attributed to Al–O stretching vibrations in AlO_4_ tetrahedra. Additionally, the band at 538 cm^−1^ shifted to a higher frequency at 583 cm^−1^ after calcination, while the Si–O–Si bending vibration remained evident at 481 cm^−1^^38,66^.

Notably, there was a gradual decrease in the intensity of the Si–O stretching band around 1100 cm^−1^, along with the broadening of Al–O–Si and Si–O–Si vibrations (790–480 cm^−1^) as the SLS content increased. This change corresponds with XRD results that show a reduction in crystalline quartz and the formation of a new aluminosilicate phase, such as albite. The weakening of the hydroxyl band near 3458 cm^−1^ suggests partial dehydroxylation and structural disorder, aligning with the presence of mullite detected across all compositions^59^. In contrast, samples containing GS exhibited sharper and more defined Si–O bands (at 1100 cm^−1^), indicating higher crystallinity, which was previously confirmed by XRD, revealing an increase in plagioclase (anorthite, albite) and quartz phases with GS addition^63^. Collectively, these findings suggest that the incorporation of SLS promotes amorphization and the formation of a glassy phase, while GS enhances the crystalline silicate framework by enriching plagioclase and quartz phases.

#### SEM analysis

**Fig. 11:**
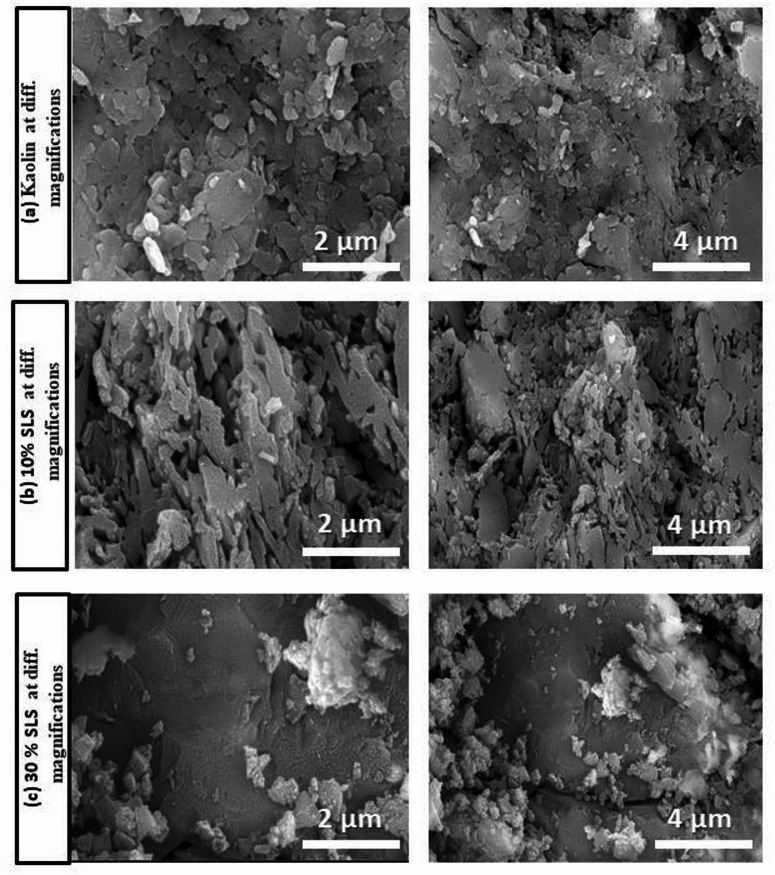
SEM micrograph of kaolin sintered at 1100 °C without and with 10 and 30% of SLS.

**Fig. 12:**
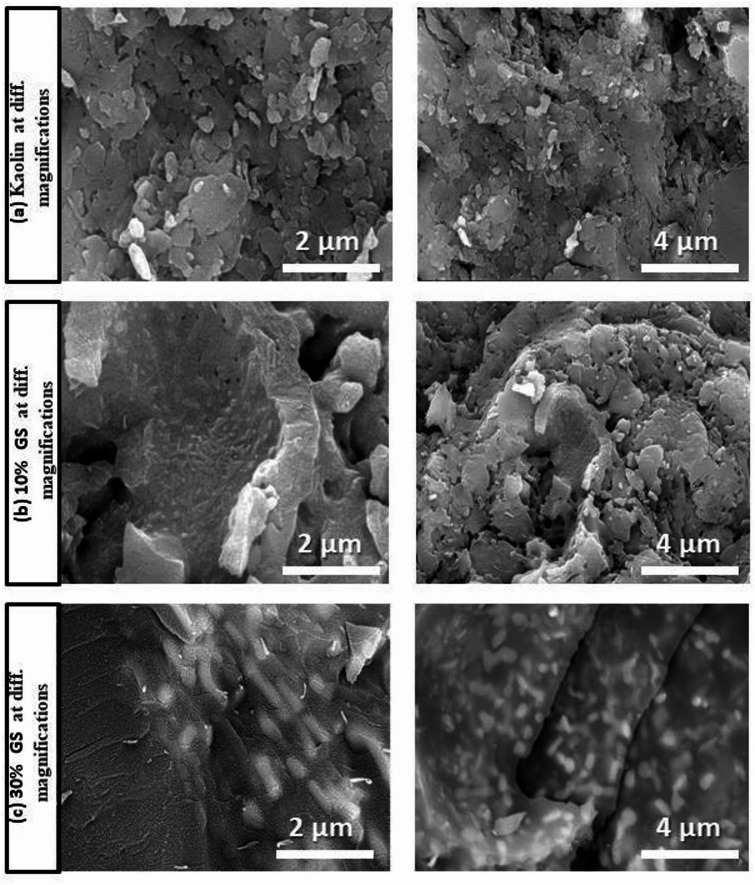
SEM micrograph of kaolin sintered at 1100 °C without and with 10 and 30% of GS.

Figures 11 and 12 show the SEM micrographs of selected samples fired at 1100 °C containing different proportions of SLS and GS, respectively. The images reveal the presence of angular quartz particles that appear only slightly dissolved. Mullite is observed as fine dot-like particles in Fig. 11b and as elongated, fibrous crystals in Fig. 12b and c. These observations are consistent with the XRD results. Mullite decreases, and a significant quantity of glassy phase and plagioclase growth are more noticeable when the amount of SLS is increased. This fills the spaces between the particles and lowers porosity as seen in Fig. 11c^50,56^. Plagioclase is shown in cluster form in Figs. 11a and 12a while in elongated white form in the sample with 30 wt.% GS (Fig. 12c). The mullite phase is promoted at lower temperatures (1100 °C), as shown by SEM and XRD. The increased formation of mullite crystals at higher temperatures in GS samples can be ascribed to the presence of Fe_2_O_3_ up to 6.76%, also present in SLS samples^67^. The development of fibrous mullite was ascribed to TiO_2_, which enhances mullite crystallization by decreasing melt viscosity, hence promoting mullite crystal nucleation and evolution^68^.

#### Mechanical properties

**Fig. 13:**
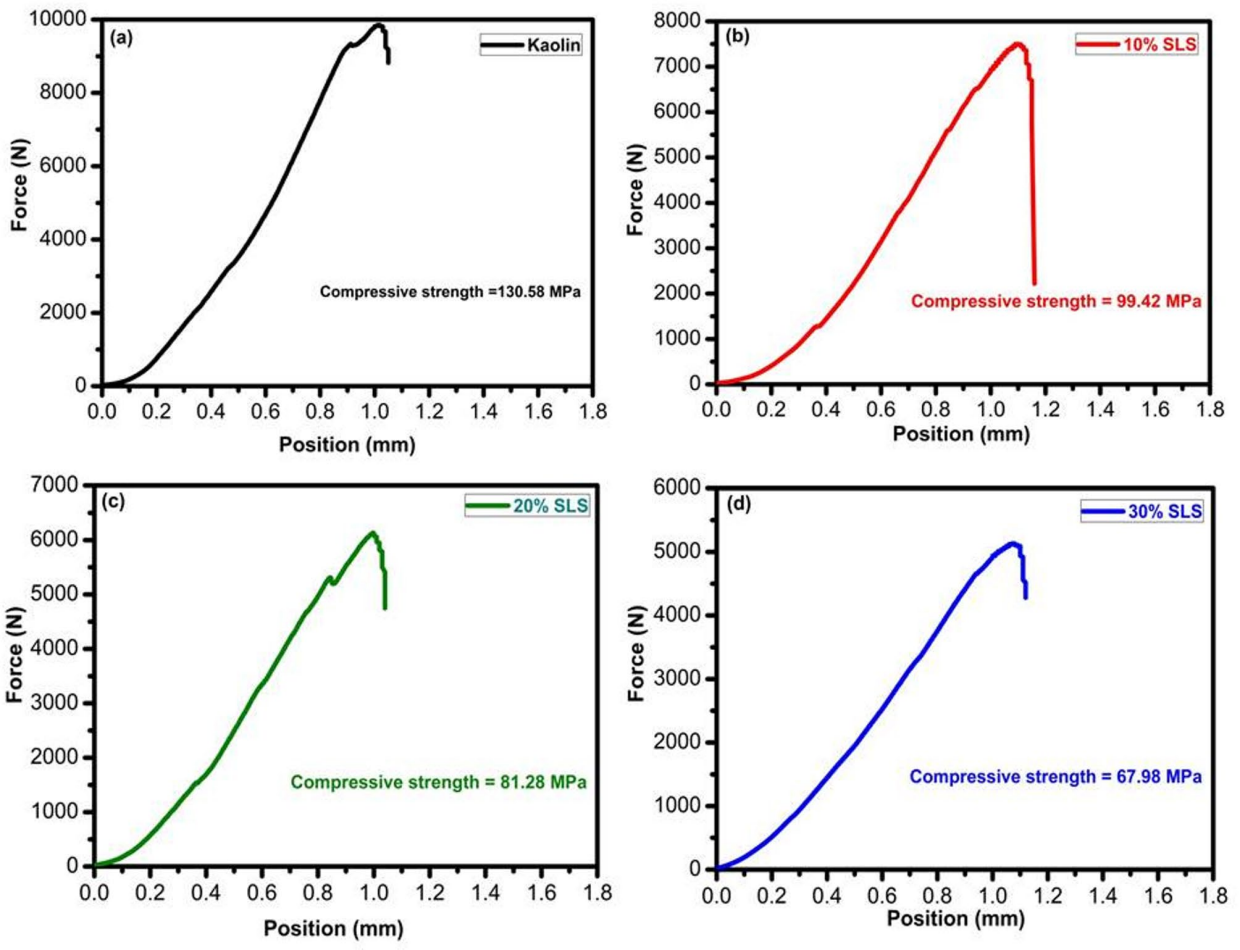
Compressive strength of kaolin sintered at 1100 °C without and with 10–30% of SLS.

**Fig. 14:**
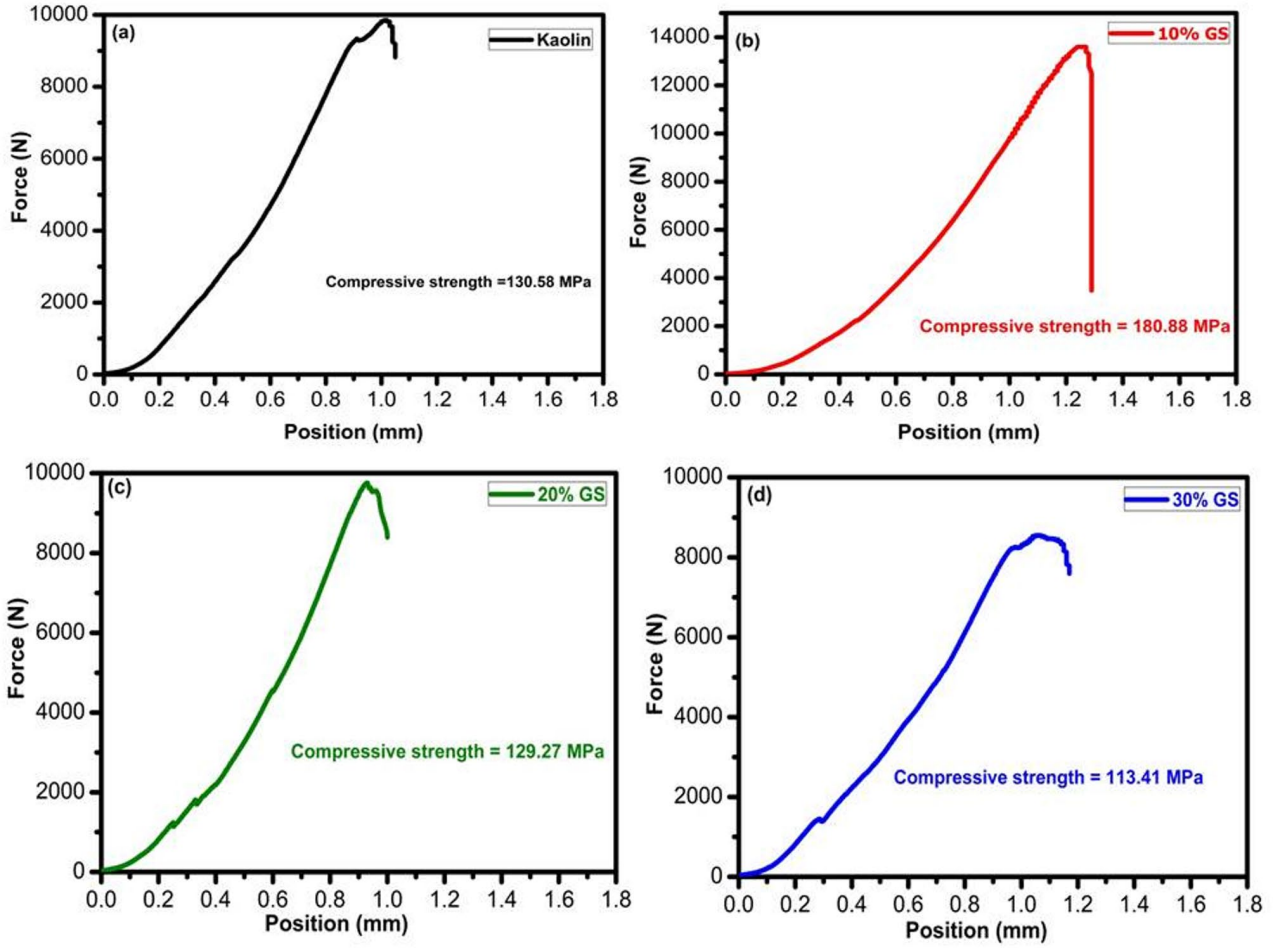
Compressive strength of kaolin sintered at 1100 °C without and with 10–30% of GS.

The compressive strength of fired samples with varying percentages of SLS and GS at 1100°C is displayed in Table 4, Figs. 13 and 14, respectively. In the present investigation, adding SLS at the expense of kaolin powder and firing it at 1100 °C resulted in a drop in strength when compared to a kaolin sample (Fig. 13 and Table 4). This is because the high flux content made the sample extremely glassy and, as a result, fragile^69,70^. This interpretation is supported by the work of Abdul Bakil et al.^70^, who reported that adding 5 wt% soda-lime silicate significantly increased the strength of stoneware bodies from 45.76 to 67.29 MPa at 1100 °C. However, further increasing the SLS content to 7 wt% and 10 wt% resulted in a reduction in strength, attributed to the excessive formation of a glassy phase within the microstructure caused by the higher soda-lime silicate content. At these higher levels, the stoneware bodies became excessively glassy and consequently more brittle. Also, these results are consistent with the findings of El-Shimy et al.^71^, who observed that an increase in the glassy phase promotes expansion and dilation of closed pores due to the entrapment of released gases—mainly CO_2_—leading to the development of microcracks within the samples. Similarly, Ozturk and Ay^72^ reported that an increased glassy phase, resulting from the higher total alkaline oxide content (Na_2_O, K_2_O, CaO) in the samples, enhanced densification, reduced open porosity, and consequently improved the firing strength.

**Table 4:** Compressive strength of kaolin sintered at 1100 °C without and with SLS and GS.

Group name	Kaolin	10%SLS	20%SLS	30%SLS	10%GS	20%GS	30%GS
Compressive strength (MPa)	130.58	99.42	81.28	67.98	180.88	129.27	113.41

A significant increase in compressive strength was observed as the GS content increased up to 10 wt.% (Fig. 14). After incorporating GS, compressive strength values at 1100 ºC ranged between 113 and 180 MPa (Table 4). The addition of varying amounts of granite sludge notably improved the compressive strength, especially at 10 wt.% GS, compared to pure kaolin. Compressive strength remains a key mechanical property for evaluating the performance of fired products. This enhancement was expected, as the feldspar minerals (albite) in the granite waste promoted densification and vitrification through the liquid phase during sintering, resulting in an adequate amount of alkaline fluxes, particularly Na_2_O and K_2_O^50,73–75^. The production of more fibrous mullite in the 10GS sample compared with the 30% GS sample may cause an increase in strength. The following is the well-known breakdown of kaolinite to metakaolinite at 650 °C^50,76^:

3 $${\text{Al}}_{{2}} {\text{O}}_{{3}} \cdot{\text{2SiO}}_{{2}} \cdot{\text{2H}}_{{2}} {\text{O }}\left( {{\text{Kaolinite}}} \right) \, \to {\text{ Al}}_{{2}} {\text{O}}_{{3}} \cdot{\text{2SiO}}_{{2}} \left( {{\text{Metakaolinite}}} \right) \, + {\text{ 2 H}}_{{2}} {\text{O}}$$

At temperatures higher than 962 °C, the latter is converted to mullite in the manner described below:

4 $${\text{Al}}_{{2}} {\text{O}}_{{3}} \cdot{\text{2SiO}}_{{2}} \left( {{\text{Metakaolinite}}} \right) \, \to \, \left( {{1}/{3}} \right) \, \left( {{\text{3Al}}_{{2}} {\text{O}}_{{3}} \cdot{\text{2SiO}}_{{2}} } \right) \, \left( {{\text{Mullite}}} \right) + \, \left( {{4}/{3}} \right){\text{SiO}}_{{2}}$$

This indicates that the mullite starts to crystallize at around 1000 °C, along with the conversion of β-quartz to α-cristobalite. At the same time, adding alkali oxides in the form of granite sludge slows down or even stops the crystallization of cristobalite, lowering the temperature at which mullite forms^77^. However, the modified kaolin samples burnt at 1100 °C showed a sharp decline in compressive strength when the proportion of granite waste (> 40 wt%) increased. Because of their volumetric variation at 573 °C (Fig. 9), the quartz content increases with the addition of granite waste, which can lead to the appearance of microcracks and a decrease in strength^71^, as shown in the SEM of the previous image (Fig. 12c). This is the reason for the reduction of compressive strength.

#### Dielectric constant properties

**Fig. 15:**
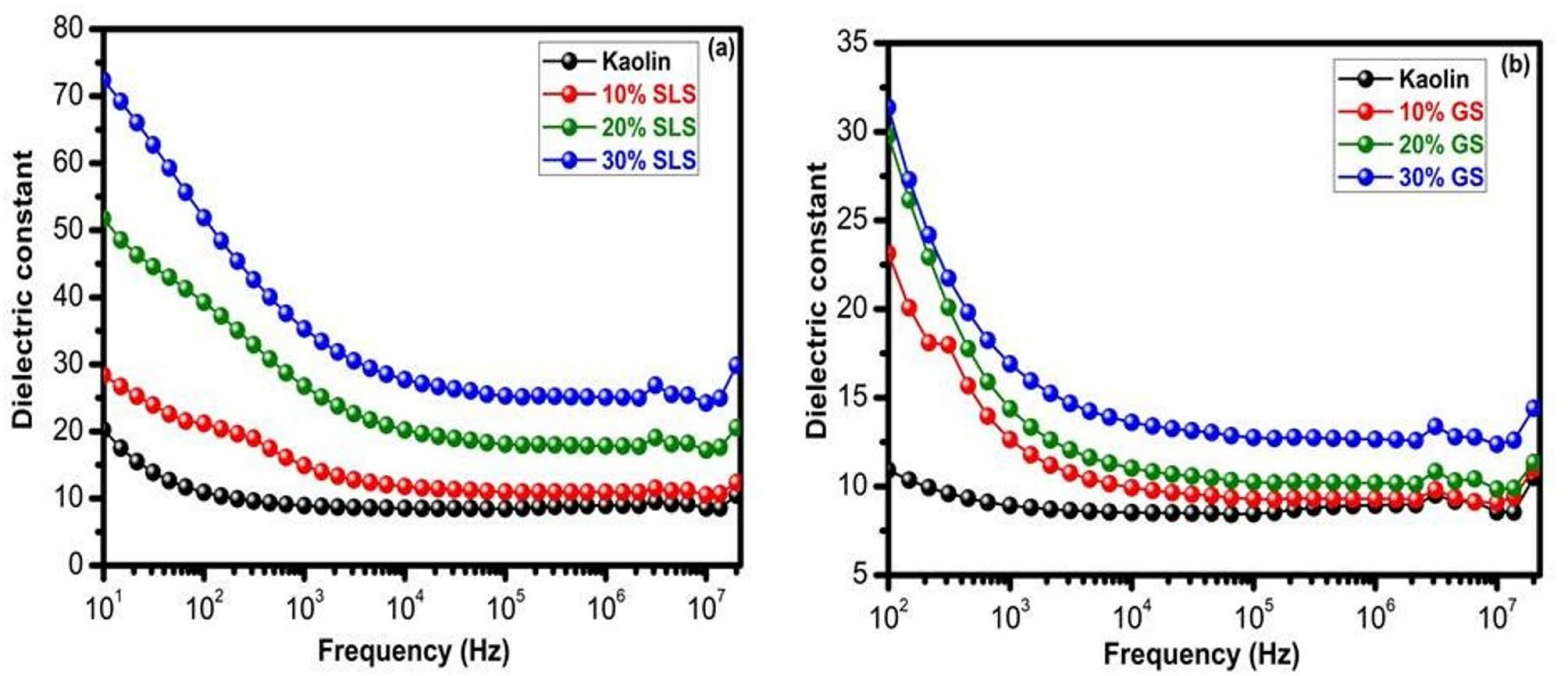
Dielectric constant of kaolin sintered at 1100 °C containing 10–30 wt.% of (**a**) SLS and (**b**) GS at different frequencies.

The dielectric constant ε′ of the fired samples with varying percentages of SLS and GS at 1100°C is shown against frequency in Fig. 15a and b, respectively. The sharp drop in dielectric constant with increasing frequency seen in the observed dispersion is consistent with the characteristics of other dielectric materials documented^78,79^. Several forms of polarization, including interfacial, ionic, and electronic, space charge, and dipolar polarization, may be responsible for the drop in dielectric constant in the high-frequency domain^80,81^. Some charge carriers are inhibited at the internal ceramic grain boundary, which causes this polarization^82,83^. It may also result from impurities or additions, which subsequently help increase this ceramic’s capacitance^84–86^. At higher frequencies, however, the quick time domain prevents the ions or dipoles from accumulating at the interface and following the field, resulting in a drop of the dielectric constant^87^.

Furthermore, it was noted that samples containing 30 weight percent SLS and GS at low frequencies had higher dielectric constants than pure kaolin or those with low concentrations of both. The impact of porosity is responsible for this. Additionally, the presence of alkali ions causes an increase in the dielectric constant with SLS and GS. It is commonly recognized that the presence of ions with greater ionic mobility in the matrix, like alkali ions, can raise the dielectric constant^88^.

#### Dielectric loss properties

**Fig. 16:**
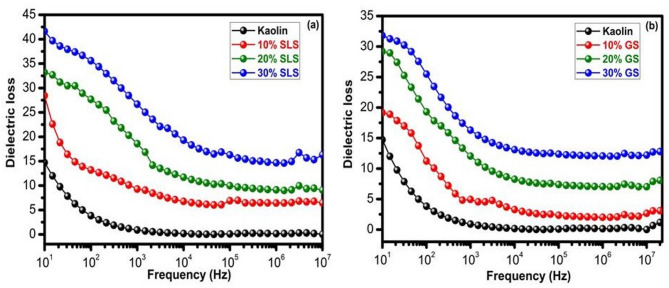
Dielectric loss of kaolin sintered at 1100 °C containing 10–30 wt.% of (**a**) SLS and (**b**) GS at different frequencies.

A plot of the dielectric loss for the fired samples with varying percentages of SLS and GS at 1100 °C as a function of frequency is displayed in Fig. 16a and b, respectively. The line graph in Fig. 16 shows that the dielectric loss is nearly constant at higher frequencies but decreases significantly in the low-frequency domain as the frequency domain increases. This pattern is similar to the frequency variation of the dielectric constant reported elsewhere^89^. As may be predicted, the phase change in the ceramic structure causes a decrease in dielectric loss. The devitrification of cullet in the ceramic matrix dominates the proportion of ceramics as the SLS and GS concentrations rise. This devitrification causes dielectric loss by producing a significant liquid phase^90–92^. The phase modification is nearly complete in the higher-frequency domain, where the loss is almost constant. All composite samples have dielectric loss values that are primarily less than one, which qualifies them for use in electronic applications. The limited variation in dielectric constant, dielectric loss, and conductivity confirms the compositional similarity of the materials consisting mainly of boromullite, amorphous silica, and a small amount of alumina with generally low electrical property values^88^.

#### Electrical conductivity

**Fig. 17:**
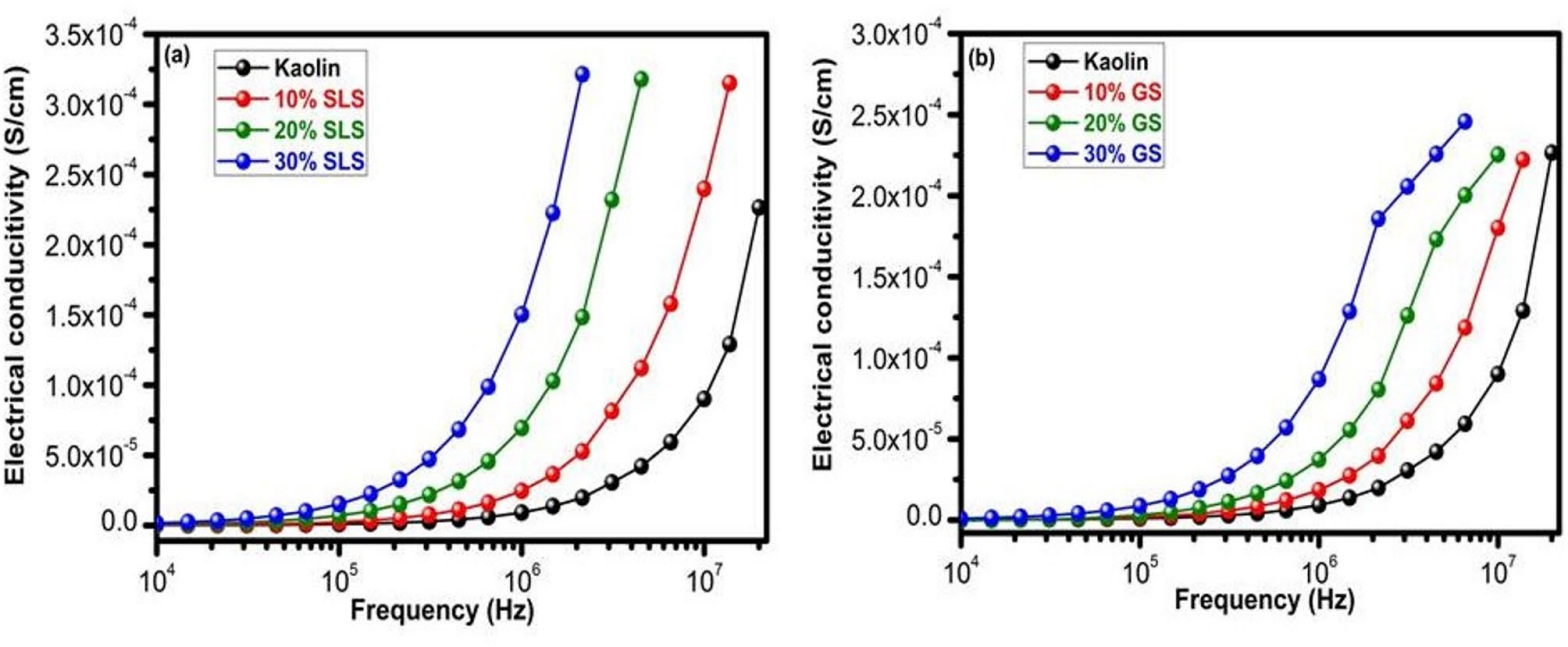
Electrical conductivity of kaolin sintered at 1100 °C containing 10–30 wt.% of (**a**) SLS and (**b**) GS at different frequencies.

Figure 17a and b display the conductivity of the fired samples at 1100 °C with different proportions of SLS and GS against frequency. In the 1 Hz to 10 MHz range, the conductivity fluctuation with the logarithm of angular frequency (lgω) was examined at room temperature. The electrical conductivity values achieved for the manufactured samples are extremely low. The frequency dependence of the sample’s conductivity indicates that conductivity increases nearly precisely as frequency increases. As the SLS and GS contents rise, the values rise as well. Compared to the GS sample, the SLS sample is more conductive. One important component of conductivity is the local migration of Na^+^ cations in wastes, which is a single hop-hopping process for hopping charges between localized states^67^. The structural makeup and the existence of insulating phases in both compositions may have an impact on their low electrical conductivity.

## Conclusion

A stable ceramic with the composition of kolain and 10–30 weight percentage of GS or SLS was successfully prepared, and its measured properties were summarized as follows:

- A densification process was successfully conducted after the addition of SLS or GS in the kaolin ceramic materials when samples were sintered at 1100 °C.
- The high alkali content in SLS reduces the porosity to 6.50% at 1100 °C in kaolin ceramics compared to GS content (16.26%). However, the smallest change in bulk density of kaolin ceramics (2.11 g/cm^3^) is observed after the addition of 30 weight percentage of SLS (2.28 g/cm^3^) or GS (2.23 g/cm^3^) at 1100 °C.
- XRD indicated the enhancement of albite contents and a decrease in quartz contents by adding SLS. On the contrary, anorthite, albite, and quartz increase by adding GS.
- Maximum strength is obtained after adding 10 weight percentage of GS to achieve 180 MPa. The strength decreases with the addition of SLS and ranges from 99 to 67 MPa.
- Dielectrical and electrical properties are enhanced by SLS addition than GS. However, both compositions have limited electrical conductivity, which may be influenced by their structural composition and the presence of insulating phases.

## Data Availability

The datasets generated and/or analyzed during the current study are not publicly available because they are private, but are available from the corresponding author on reasonable request.
